# Wideband Circularly Polarized Conformal Antenna with Physics-Informed Neural Network Modeling for IoBNT Capsule Endoscopy

**DOI:** 10.3390/bioengineering13060620

**Published:** 2026-05-26

**Authors:** Pariya Nasirishehni, Mohammad (Behdad) Jamshidi, Mehdi Mehranpour

**Affiliations:** 1 Faculty of Engineering and Technology, Imam Khomeini International University (IKIU), Qazvin 34148-96818, Iran; parianasirishehni@gmail.com; 2 Faculty of Engineering and Information Technology, University of Technology Sydney (UTS), Sydney, NSW 2007, Australia; 3 Research and Development Department, MetaMax, Sydney, NSW 2010, Australia; 4 Department of Electrical and Computer Engineering, University of Mohaghegh Ardabili (UMA), Ardabil 56199-11367, Iran; mehranpour.mehdi@gmail.com

**Keywords:** artificial intelligence (AI), physics-informed neural network (PINN), Internet of Bio-Nano Things (IoBNT), biotechnology, capsule endoscopy, conformal microstrip antenna, circular polarization, wideband antenna

## Abstract

The convergence of artificial intelligence, biotechnology, and the Internet of Bio-Nano Things (IoBNT) is enabling the creation of a new generation of intelligent in-body medical devices for continuous diagnosis and monitoring. In this context, a compact, wideband, circularly polarized conformal microstrip antenna is proposed for capsule endoscopy applications. The antenna is integrated along the inner wall of a 10 mm-diameter capsule and achieves an impedance bandwidth of 2.06–5.39 GHz (89.39%), maintaining stable matching under varying biological tissue conditions. A 3 dB axial ratio bandwidth (ARBW) of 2.31–3.14 GHz (30.45%) ensures reliable circular polarization and robust wireless communication in lossy and dynamic in-body environments. To extend beyond conventional electromagnetic analysis, a physics-informed neural network (PINN) framework is introduced to model the thermal response of biological tissues based on the governing bioheat equation. This AI-driven approach enables fast and generalizable prediction of temperature rise under varying operational conditions without repeated numerical simulations. At 2.45 GHz, the antenna exhibits a maximum gain of −31.1 dBi with a radiation efficiency of approximately −34 dB, consistent with in-body propagation constraints. Simulation and experimental results in realistic tissue phantoms, including muscle, small intestine, large intestine, and stomach, confirm stable wideband and polarization performance. Specific absorption rate (SAR) analysis demonstrates compliance with IEEE C95.1-2019 safety limits, while link budget evaluation validates reliable telemetry over a 1–3 m communication range. The integration of advanced antenna design with physics-informed machine learning provides a scalable framework for intelligent, safe, and adaptive IoBNT-enabled capsule endoscopy systems.

## 1. Introduction

Recent advances in biomedical engineering, wireless communications, and artificial intelligence are accelerating the development of smart ingestible medical devices capable of real-time diagnosis, monitoring, and personalized healthcare. Among these technologies, wireless capsule endoscopy (WCE), first introduced as a swallowable imaging platform for gastrointestinal inspection, has transformed non-invasive visualization of the digestive tract by eliminating the discomfort and limitations associated with conventional wired endoscopy [1]. Modern capsule systems are increasingly expected to move beyond passive imaging toward intelligent sensing nodes that support bidirectional telemetry, adaptive operation, and continuous physiological monitoring. This evolution aligns closely with the emerging paradigm of the Internet of Bio-Nano Things (IoBNT), where interconnected bio-integrated devices communicate within and around the human body to enable next-generation digital healthcare ecosystems [2,3].

Despite their promise, reliable wireless communication for capsule endoscopy remains a major engineering challenge. The severe size constraints of ingestible capsules, typically around 10–11 mm in diameter, impose strict limitations on antenna geometry, available ground plane, and radiation aperture. In addition, electromagnetic waves propagating through biological tissues experience high dielectric loading, impedance detuning, absorption loss, polarization distortion, and multipath scattering due to the heterogeneous gastrointestinal environment [4,5]. These issues become more pronounced as the capsule traverses different organs such as the stomach, small intestine, and colon, each possessing distinct dielectric characteristics. Therefore, the design of compact antennas with stable wideband impedance matching, robust polarization behavior, and acceptable radiation efficiency under dynamic tissue conditions remains a critical requirement for practical WCE systems.

Extensive research has been conducted on implantable and capsule antennas to address these challenges. Miniaturized loop, folded inverted-F, and conformal antennas have demonstrated improved compactness and radiation diversity for capsule applications [6,7]. More recently, conformal MIMO and ultra-wideband telemetry structures have been investigated to enhance data throughput and communication reliability [8,9]. Other studies have explored quad-band and wideband implantable antennas for deep-tissue monitoring and wireless power transfer [10,11,12]. Circular polarization (CP) has also attracted growing attention because it mitigates polarization mismatch caused by capsule rotation and orientation randomness inside the gastrointestinal tract. Compact circularly polarized capsule antennas have shown promising results; however, many existing designs still suffer from narrow axial-ratio bandwidth, limited impedance bandwidth, structural complexity, or insufficient robustness under varying tissue environments [13,14]. In addition to homogeneous tissue phantoms, multilayer anatomical models consisting of skin, fat, muscle, and gastrointestinal tissues are widely used for realistic SAR and propagation analysis in implantable and ingestible biomedical systems. Such models better capture the dielectric discontinuities, tissue heterogeneity, and localized absorption effects encountered during practical capsule operation.

In parallel with antenna innovation, artificial intelligence is reshaping the modeling and optimization of biomedical devices. Conventional full-wave electromagnetic and thermal simulations are computationally expensive, especially when repeated across multiple tissue scenarios, material uncertainties, and safety conditions. Physics-informed neural networks (PINNs) have recently emerged as a powerful scientific machine learning framework that embeds governing differential equations directly into neural network training, enabling data-efficient and physically consistent surrogate models. PINNs have shown strong potential in biomedical digital twins, thermal analysis, and multi-physics systems where real-time prediction and generalization are essential [15,16]. In the context of capsule endoscopy, PINNs can provide rapid estimation of tissue heating governed by the bioheat equation, reducing dependence on repeated numerical simulations while supporting adaptive safety-aware operation. Motivated by these needs, this paper proposes a wideband circularly polarized conformal antenna integrated along the inner wall of a compact capsule platform and augmented with a PINN-based thermal modeling framework for IoBNT-enabled capsule endoscopy. By combining advanced antenna engineering with physics-guided artificial intelligence, the proposed system aims to improve communication robustness, patient safety, and computational efficiency in future intelligent ingestible devices.

Despite these advances, existing wireless capsule endoscopy antennas often involve trade-offs among miniaturization, bandwidth, polarization purity, radiation stability, and capsule integration. Many compact designs remain narrowband or linearly polarized, whereas some circularly polarized structures require larger form factors or increased geometrical complexity. Furthermore, simultaneously preserving wideband impedance matching and stable circular polarization inside a highly lossy and spatially heterogeneous gastrointestinal environment remains a significant challenge. Motivated by these limitations, this work proposes a compact conformal microstrip antenna integrated within a 10 mm-diameter capsule platform, providing wideband and circularly polarized performance suitable for in-body operation. In addition, a physics-informed neural network (PINN) framework is employed to model tissue thermal response through the bioheat equation, enabling the efficient estimation of temperature rise without repeated full-wave or multiphysics simulations. The main contributions of this work are summarized as follows:

- A compact conformal circularly polarized microstrip antenna is developed for a 10 mm-diameter capsule endoscopy platform with efficient utilization of the available internal volume.
- Wide impedance bandwidth and stable axial-ratio performance are achieved, supporting robust wireless telemetry under capsule rotation and varying tissue loading conditions.
- A PINN-based thermal modeling framework is introduced for rapid prediction of tissue temperature rise under different operational scenarios using the governing bioheat equation.
- The antenna performance is evaluated in realistic biological tissue phantoms including muscle, stomach, small intestine, and large intestine environments.
- Specific absorption rate (SAR) compliance and link-budget analyses are presented to assess biological safety and practical communication capability.
- The combined antenna and AI-based modeling framework provides a foundation for future intelligent IoBNT-enabled capsule endoscopy systems.

The remainder of this paper is organized as follows. Section 2 describes antenna design and configuration. Section 3 presents the simulation and measurement results. Section 4 provides the SAR analysis and biological safety evaluation and analyzes the link budget. Section 5 presents the physics-informed neural network framework for thermal prediction. Section 6 provides a comparative study with published works. Finally, Section 7 concludes the paper.

## 2. Antenna Design and Configuration

### 2.1. Substrate and Geometry

**Figure 1:**
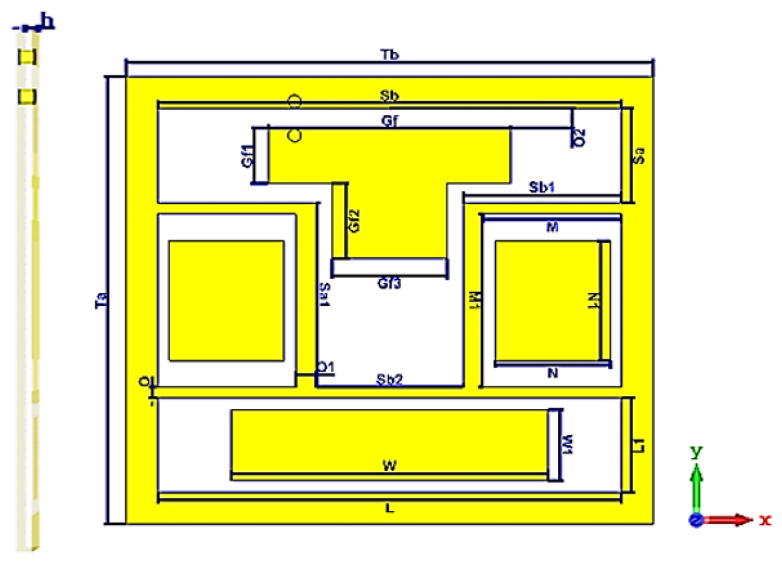
Planar schematic of the proposed conformal microstrip antenna (left: conformally wrapped on capsule rod; right: flat layout with labeled dimensions).

Figure 1 illustrates the planar radiating layer of the designed capsule antenna. The classical microstrip resonance relation can approximately describe the resonant behavior of the proposed conformal antenna:

(1) $$f_{r}\approx \frac{c}{2L_{\mathrm{eff}}\sqrt{\varepsilon_{\mathrm{eff}}}}$$

where $L_{\mathrm{eff}}$ is the effective current path length and $\varepsilon_{\mathrm{eff}}$ is the effective dielectric constant considering both the substrate and surrounding biological tissues. Due to the high permittivity of human tissues, the effective wavelength is significantly shortened, enabling antenna miniaturization within the capsule volume. Circular polarization is achieved by generating two orthogonal current components with approximately equal amplitude and quadrature phase difference through the asymmetric CPW excitation and T-shaped slot perturbations. The dual-resonance behavior introduced by the rectangular loop and slot loading further contributes to the ultra-wide impedance bandwidth and stable axial-ratio performance. The antenna is designed on a Rogers RO5880 substrate with a thickness of h=0.254 mm, a relative permittivity of $\varepsilon_{r}=2.2$, and a loss tangent of tanδ=0.0009. This low-loss, low-permittivity substrate minimizes dielectric losses that would otherwise degrade radiation efficiency in the already-lossy in-body environment. The 2.45 GHz ISM band was selected because it provides a practical compromise among antenna miniaturization, tissue penetration, regulatory availability, and telemetry bandwidth. The proposed ultra-wide impedance bandwidth further improves robustness against dielectric detuning caused by tissue variability, capsule rotation, and anatomical heterogeneity encountered during gastrointestinal transit.

The antenna topology was developed through iterative parametric optimization beginning from a conformal rectangular loop radiator. T-shaped perturbation slots and asymmetric CPW excitation were subsequently introduced to generate orthogonal current modes with quadrature phase difference, enabling wideband circular polarization while maintaining compact capsule-compatible dimensions. The conductor pattern consists of a rectangular loop, a T-shaped strip, and three rectangular strips positioned on both sides and the bottom of the antenna. The antenna is excited via a 50-Ω coplanar waveguide (CPW) feed. The T-shaped strip and the CPW gap are precisely dimensioned to control impedance matching and circular polarization generation. Strong adhesion of the metal layer to the capsule inner wall is ensured by feeding the antenna through two metallized vias from the back of the substrate.

### 2.2. Capsule Assembly and Phantom Model

**Figure 2:**
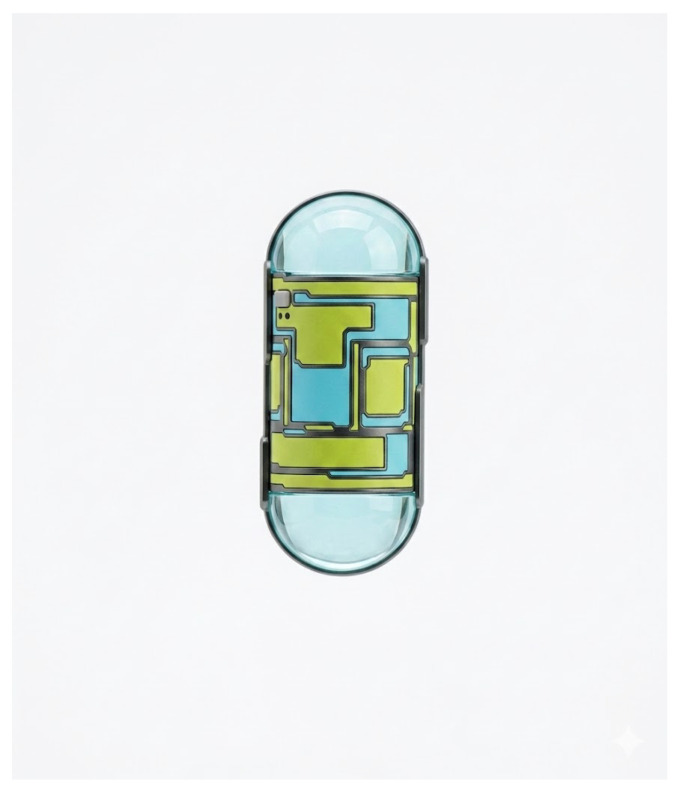
Capsule antenna at the center of a $100\times 100\times 100\;{\mathrm{mm}}^{3}$ muscle-mimicking phantom.

The planar structure is wrapped around a cylinder to fit inside a capsule with a 10 mm inner diameter, as shown in Figure 2. The capsule shell is fabricated from acrylic ($\varepsilon_{r}=3.0$, tanδ=0.001) with a wall thickness of 0.5 mm. This thin shell provides mechanical protection while introducing only minor electromagnetic perturbation, as verified through parametric simulation. Figure 2 shows the simulation phantom: a single-layer muscle-mimicking cube (100mm×100mm×100mm) with the capsule at the center. The dielectric properties of muscle tissue at 2.45 GHz are modeled as $\varepsilon_{r}=52.73$ and σ=1.74 S/m, based on the Gabriel tissue parameter database [17]. All simulations are performed in CST Microwave Studio with a time-domain solver and adaptive mesh refinement. Beyond telemetry, the proposed conformal platform may also support future biosensing functionality by monitoring impedance perturbations or dielectric variations associated with localized physiological changes.

### 2.3. Antenna Dimensions

The optimized antenna dimensions are summarized in Table 1. Key parameters include the T-shaped slot dimensions ($S_{a}$, $S_{b}$, $S_{b1}$, $S_{b2}$) which govern the CP mechanism, and the CPW gap parameters ($G_{f1}$, $G_{f2}$, $G_{f3}$) which determine impedance matching.

## 3. Results and Discussion

### 3.1. Reflection Coefficient ($S_{11}$)

**Figure 3:**
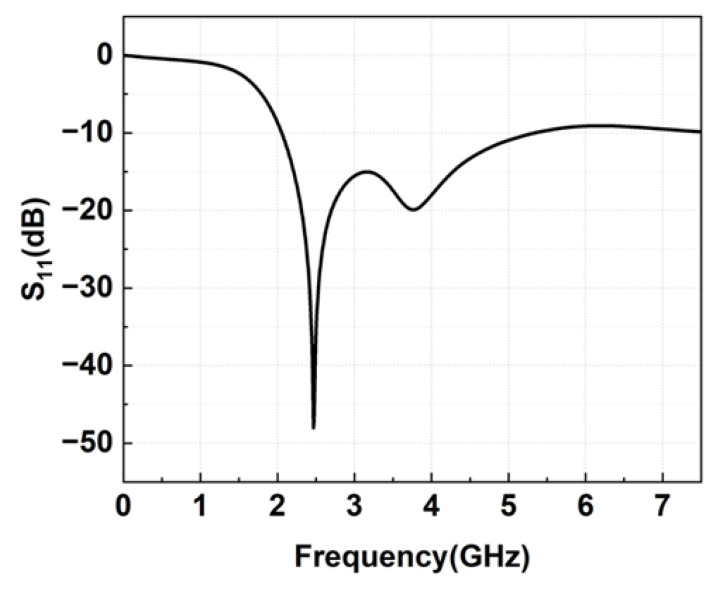
Simulated and measured $S_{11}$ of the proposed antenna in muscle phantom, showing dual resonances at 2.49 GHz and 3.80 GHz with a −10 dB bandwidth of 89.39%.

The simulated and measured $S_{11}$ results are presented in Figure 3. Both curves exhibit excellent agreement, confirming the accuracy of the CST-based design. $S_{11}$ remains below −10 dB across 2.06–5.39 GHz, corresponding to a fractional bandwidth of 89.39%. This ultra-wideband (UWB) performance fully covers the 2.45 GHz ISM band and provides substantial margins for frequency shifts induced by tissue variability. Two dominant resonance frequencies are observed at 2.49 GHz and 3.8 GHz, arising from the combined effects of the T-shaped structure, its internal slots, and the lower rectangular strip. These dual resonances underpin the exceptionally wide impedance bandwidth of the design.

### 3.2. Surface Current Distribution

**Figure 4:**
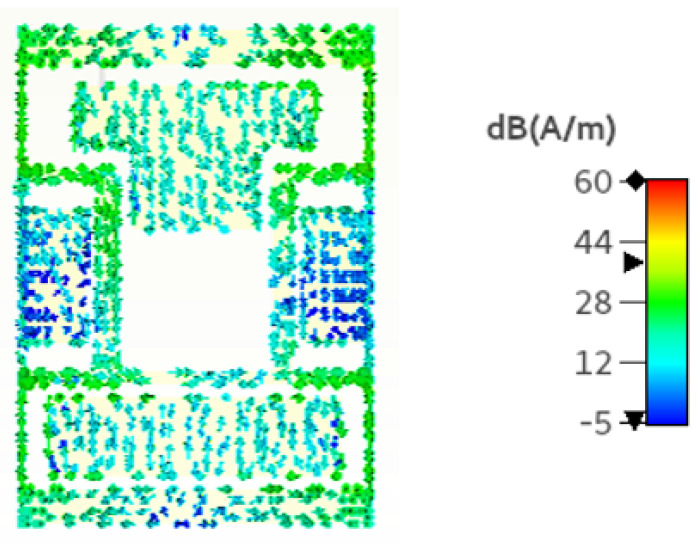
Simulated Surface current distributions at 90° phase. The color scale indicates the magnitude of the surface current density in dB(A/m), ranging from red (highest current density) to blue (lowest current density).

Figure 4 presents the simulated surface current distributions at 90° phase for both resonance frequencies. At 2.49 GHz, the current concentrates along the rectangular loop and T-strip edges. At 3.8 GHz, the current activity shifts toward the upper T-shaped slot region, confirming dual-mode excitation responsible for the wideband response.

### 3.3. Circular Polarization and Axial Ratio

**Figure 5:**
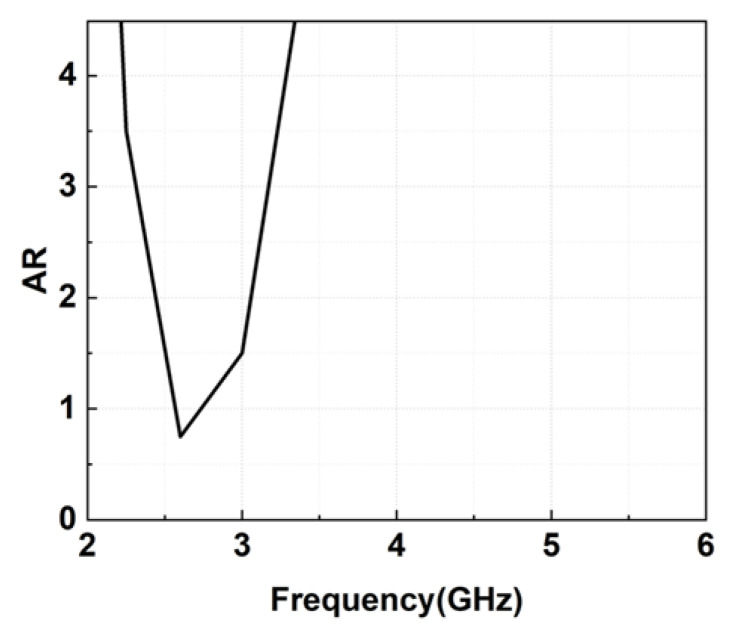
Simulated and measured 3-dB axial ratio. The ARBW of 2.31–3.14 GHz (30.45%) overlaps well with the impedance bandwidth.

**Figure 6:**
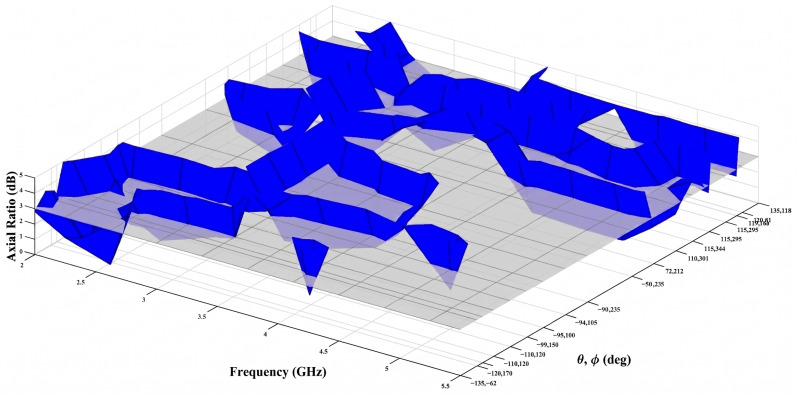
Broadband 3D axial ratio distribution (MATLAB) across 2–5.5 GHz. Shaded region indicates AR < 3 dB within ±135°, confirming stable LHCP radiation.

The proposed antenna generates CP through asymmetric CPW feeding combined with two T-shaped slots and a bottom rectangular slot. Proper adjustment of $S_{a}$ and $S_{b}$ produces two orthogonal electric field components with a 90° phase difference. The time-varying surface current distributions at 0°, 90°, 180°, and 270° phase angles confirm clockwise current rotation with increasing phase, indicating left-hand circular polarization (LHCP) in the +Z direction. The axial ratio (AR) performance is presented in Figure 5. The optimized 3 dB ARBW extends from 2.31 GHz to 3.14 GHz (30.45% fractional bandwidth). The measured AR reaches 1.8 dB at 2.45 GHz and remains below 0.46 dB at 2.62 GHz, confirming stable CP at θ=100 and φ=−93. The wide overlap between the impedance bandwidth and the ARBW ensures consistent CP performance across the entire operational range. Figure 6 illustrates the spatial stability of circular polarization over frequency and angular directions. The shaded region with AR < 3 dB confirms that stable LHCP radiation is preserved across a wide angular sector, reducing polarization mismatch caused by random capsule orientation.

Figure 6 presents a MATLAB-generated broadband AR distribution across 2–5.5 GHz. Within ±135°, the AR remains below 3 dB (shaded region), confirming stable LHCP radiation in the main radiation directions and across a wide frequency span.

### 3.4. Radiation Patterns

**Figure 7:**
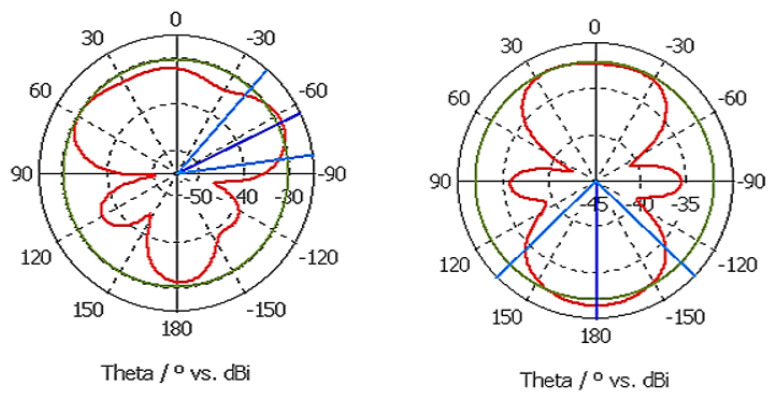
Simulated measured normalized radiation patterns at 2.45 GHz in the XZ-plane and YZ-plane inside the muscle phantom.

The radiation patterns at 2.45 GHz are depicted in Figure 7. Simulated and measured characteristics in the XZ and YZ planes show similar behavior. The antenna exhibits a maximum gain of −31.1 dBi and a radiation efficiency of approximately −34.31 dB. Such low gain and efficiency values are expected for in-body biomedical antennas due to strong electromagnetic energy absorption by surrounding biological tissues. For in-body capsule telemetry systems, realized gain values are typically much lower than free-space antennas because biological tissues strongly attenuate electromagnetic radiation. Therefore, practical communication capability should be evaluated together with link-budget margin and telemetry reliability rather than isolated gain values.Although the realized gain and radiation efficiency are low in absolute terms, such values are characteristic of deeply embedded biomedical antennas operating in highly lossy tissues. The positive link margin confirms that reliable telemetry remains feasible despite severe propagation attenuation.

### 3.5. Tissue Environment Robustness

**Figure 8:**
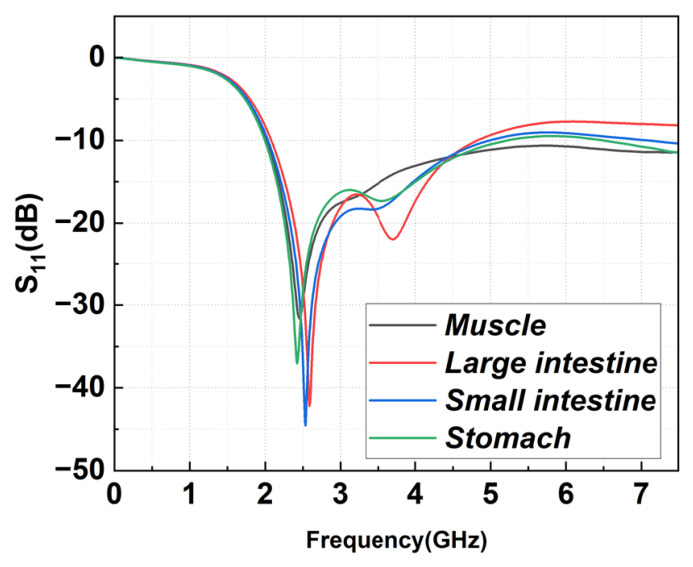
Simulated $S_{11}$ in four body tissue environments (muscle, large intestine, small intestine, stomach), demonstrating robust wideband stability across GI tract conditions.

A critical challenge for in-body antennas is maintaining stable impedance matching as the capsule traverses different GI tissue segments. Figure 8 presents the simulated $S_{11}$ in four distinct tissue environments: muscle ($\varepsilon_{r}=52.73$, σ=1.74 S/m), large intestine ($\varepsilon_{r}=59.7$, σ=1.03 S/m), small intestine ($\varepsilon_{r}=58.4$, σ=1.63 S/m), and stomach ($\varepsilon_{r}=66.2$, σ=1.99 S/m) at 2.45 GHz [17]. In all cases, $S_{11}$ remains below −10 dB across the full operating band, with only minor frequency shifts, demonstrating the design robustness afforded by the ultra-wide impedance bandwidth.

## 4. SAR Analysis and Biological Safety

**Figure 9:**
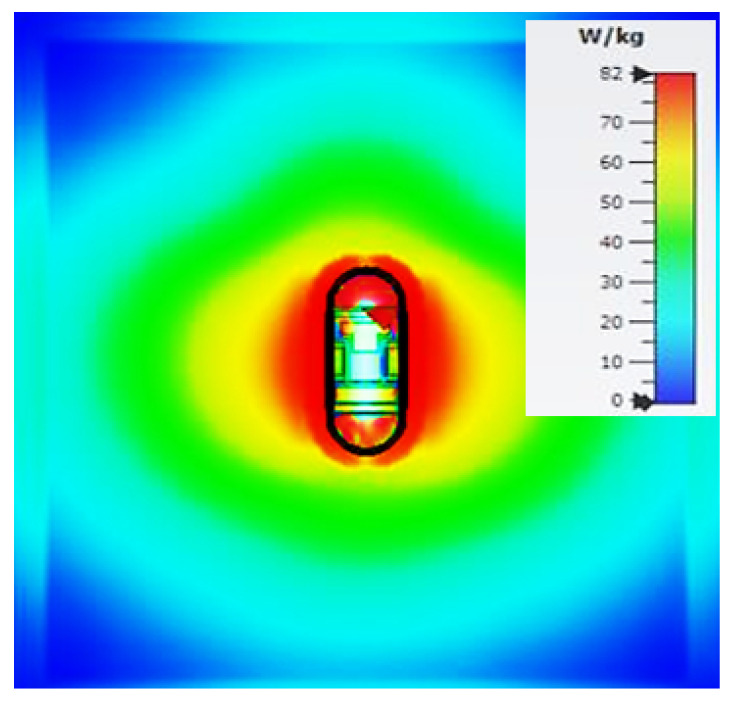
Simulated SAR distribution at 2.45 GHz. The color scale represents SAR distribution in W/kg, where red indicates the maximum energy absorption zone (up to 82 W/kg) concentrated near the capsule, and blue denotes areas of minimal or zero electromagnetic energy absorption.

Specific absorption rate (SAR) is one of the most important safety indicators for wireless implantable and ingestible biomedical devices, as it quantifies the rate at which electromagnetic energy is absorbed by surrounding biological tissues. In IoBNT-enabled capsule endoscopy systems, where miniature devices operate inside the gastrointestinal tract for sensing, imaging, and telemetry, maintaining low SAR is essential to avoid excessive localized tissue heating and to ensure safe long-duration operation. Since the proposed capsule antenna radiates in close proximity to lossy internal organs, SAR evaluation is required to verify compliance with international human exposure regulations. Figure 9 illustrates the simulated SAR distributions of the proposed capsule antenna at the 2.45 GHz ISM band using standard 1 g and 10 g tissue averaging masses. The highest energy absorption is concentrated in the tissue region immediately surrounding the capsule surface, while the absorbed power decays rapidly with distance due to tissue attenuation and near-field dissipation. This behavior is expected for low-power in-body telemetry devices and indicates that the electromagnetic exposure remains strongly localized around the capsule.

For the 1 g averaging mass, the maximum SAR is 0.82 W/kg, which is substantially below the IEEE C95.1-2019 [18] exposure limit of 1.6 W/kg. Based on this result, the equivalent allowable input power can reach approximately 19.51 mW while remaining within the IEEE safety threshold. For the 10 g averaging mass, the maximum SAR is 0.263 W/kg, which is also well below the ICNIRP limit of 2.0 W/kg, corresponding to an allowable input power of approximately 76.04 mW. In practical operation, the proposed capsule transmitter is intended to work at a very low output power of approximately −4 dBm (0.39 mW), which is significantly lower than both calculated safety margins. Therefore, the actual operating condition provides a wide safety buffer relative to regulatory limits. This low-power characteristic is highly desirable for IoBNT medical nodes because it simultaneously reduces tissue exposure, extends battery lifetime, and supports continuous wireless monitoring. Electromagnetic propagation inside biological tissues is strongly affected by dielectric losses, conductivity, scattering, and near-field absorption. Consequently, wideband circular polarization and stable impedance matching become critical for maintaining reliable WBAN and IoBNT telemetry links in dynamic in-body environments.

Compared with conventional SAR-only analyses [19], the proposed framework further incorporates a physics-informed neural network capable of rapidly predicting tissue temperature rise without repeated numerical simulations, thereby. Overall, enabling computationally efficient safety-aware operation. The SAR analysis was conducted under near-field conditions because the proposed antenna operates directly inside lossy biological tissues where reactive and radiating near-field interactions dominate electromagnetic energy absorption. Overall, the SAR results confirm that the proposed circularly polarized conformal capsule antenna can provide reliable in-body wireless communication while satisfying recognized international safety standards. These findings demonstrate the suitability of the proposed platform for next-generation intelligent capsule endoscopy and other IoBNT biomedical sensing applications requiring safe, miniature, and energy-efficient wireless devices.

A link budget analysis was conducted to assess wireless communication capability between the implantable capsule antenna and an external omnidirectional CP base station antenna using the Friis free-space transmission model. The key parameters and results are summarized in Table 2. The implantable antenna operates with a transmission power of −40 dBW (10 μW) and a gain of −31.1 dBi, while the receiving antenna provides a gain of 2.15 dBi. For a communication distance of 1–3 m, the free-space path loss ranges from 40.25 dB to 49.79 dB. The calculated $C_{0}/N_{0}$ of 81.21–90.75 dB/Hz exceeds the required threshold of 79.09 dB/Hz, yielding a positive link margin of 2.1–11 dB. These results confirm a stable and efficient wireless telemetry link suitable for capsule endoscopy in a clinical setting.

## 5. Physics-Informed Neural Network for Thermal Dose Prediction

### 5.1. Motivation and Overview

The proposed PINN framework complements the antenna subsystem by enabling rapid thermal safety estimation under varying operating conditions without repeated computationally expensive multiphysics simulations. This integration supports future adaptive and safety-aware IoBNT capsule systems. Conventional numerical solvers such as finite differences (FD) produce accurate solutions to the Pennes bioheat equation but must be re-run for every new input (tissue type, transmit power, capsule geometry). A physics-informed neural network (PINN) [20,21] embeds the governing differential equation directly into the training loss, producing a mesh-free surrogate that satisfies the physics at any queried point without re-solving the full linear system. This section formulates the PINN, derives the coordinate substitution that enables stable convergence, and presents three analytical figures that together characterise the capsule antenna’s bandwidth robustness and thermal safety across all four GI tissue environments.

### 5.2. Bandwidth Sensitivity Across GI Tissue Environments

**Figure 10:**
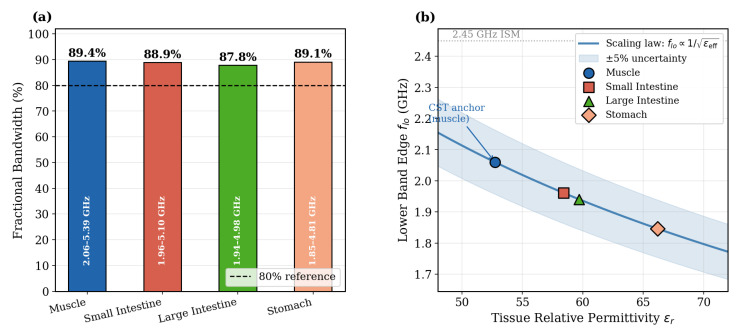
Analytical prediction of tissue-dependent impedance bandwidth anchored to the CST-simulated muscle result (89.4%). (**a**) Fractional bandwidth for each GI tissue environment predicted by the quasi-static scaling law (Equation (2)); band edges $\left[f_{\mathrm{lo}},\;f_{\mathrm{hi}}\right]$ are printed inside each bar. All four tissues exceed 87% FBW, confirming that the wideband design is robust to the dielectric loading variation encountered along the GI tract. (**b**) Continuous scaling-law curve of lower band edge $f_{\mathrm{lo}}$ vs. tissue relative permittivity $\varepsilon_{r}$; shaded band indicates ±5% model uncertainty.

As a wireless capsule endoscope (WCE) traverses the gastrointestinal (GI) tract, the surrounding dielectric environment shifts substantially between tissues such as muscle, stomach, small intestine, and colon [17]. Because the resonant frequency of a microstrip patch antenna scales as $f_{r}\propto 1/\sqrt{\varepsilon_{\mathrm{eff}}}$ [22,23], where $\varepsilon_{\mathrm{eff}}=\left(\varepsilon_{\mathrm{sub}}+\varepsilon_{r}\right)/2$ is the effective permittivity seen by the guided wave, these tissue-to-tissue permittivity variations directly detune the antenna and compress its impedance bandwidth [24]. Quantifying this sensitivity before introducing the physics-informed thermal solver establishes the robustness requirements the antenna must satisfy throughout a full endoscopic transit. The resonant frequency of a patch antenna scales as $f_{r}\propto 1/\sqrt{\varepsilon_{\mathrm{eff}}}$, where $\varepsilon_{\mathrm{eff}}=\left(\varepsilon_{\mathrm{sub}}+\varepsilon_{r}\right)/2$ is the effective permittivity seen by the guided wave. Using the CST-simulated muscle result ($f_{\mathrm{lo}}=2.06$ GHz, $f_{\mathrm{hi}}=5.39$ GHz, FBW=89.4%) as the anchor, the lower band edge for any other tissue is predicted by

(2) $$f_{\mathrm{lo}}\left(\varepsilon_{r}\right)=f_{\mathrm{lo}}^{\mathrm{ref}}\sqrt{\frac{\varepsilon_{\mathrm{eff}}^{\mathrm{ref}}}{\varepsilon_{\mathrm{eff}}\left(\varepsilon_{r}\right)}},$$

and the upper edge is adjusted for the conductivity-dependent loss tangent. Figure 10 shows the results. Figure 10a plots the predicted fractional bandwidth (FBW) for each of the four tissue environments. All four values exceed 87%, with muscle anchored at the CST-simulated 89.4% and the remaining tissues falling within a 1.6 percentage-point band. The band edges $\left[f_{\mathrm{lo}},\;f_{\mathrm{hi}}\right]$ printed inside each bar confirm that the 2.45 GHz ISM channel remains well inside the operational window for every tissue. Figure 10b traces the continuous scaling-law prediction of $f_{\mathrm{lo}}$ against tissue permittivity $\varepsilon_{r}$ over the physiological range 48–72. The ±5% uncertainty band (shaded) accounts for manufacturing tolerances in the Rogers RO5880 substrate. All four tissue operating points fall within this band, confirming the internal consistency of the quasi-static model.

### 5.3. Governing Equation

The steady-state Pennes bioheat equation in spherical symmetry is

(3) $$\frac{d^{2}u}{dr^{2}}+\frac{2}{r}\;\frac{du}{dr}-\alpha^{2}\;u=-Q\left(r\right),\;r\in \left[r_{0},\;r_{\infty }\right],$$

where $u\left(r\right)=T\left(r\right)-T_{\mathrm{body}}$ is the temperature rise [°C], $\alpha^{2}=\omega_{b}c_{b}/k=31.92$ m^−2^ is the blood-perfusion coefficient, and Q(r) is the electromagnetic heat source

(4) $$Q\left(r\right)=\frac{\rho \;{\mathrm{SAR}}_{1\;W}}{k}{\left(\frac{r_{0}}{r}\right)}^{\;2}\exp\;\left(-\frac{2(r-r_{0})}{\delta }\right),$$

where δ=22.3 mm is the electromagnetic skin depth in muscle tissue (from $\varepsilon_{r}=52.73$, σ=1.74 S/m at 2.45 GHz), and $r_{0}=5$ mm is the capsule radius. The boundary conditions are

(5) $$\begin{array}{cccc} \frac{du}{dr}{|}_{r=r_{0}} & =0\; & \; & (\mathrm{Neumann}:\;\mathrm{thermally}\;\mathrm{insulated}\;\mathrm{capsule}\;\mathrm{surface}), \end{array}$$

(6) $$\begin{array}{cccc} \;u(r_{\infty }) & =0\; & & \;\;(\mathrm{Dirichlet}:\;\mathrm{body-core}\;\mathrm{temperature}\;\mathrm{at}\;r_{\infty }=70\;\mathrm{mm}). \end{array}$$

### 5.4. Implementation and Numerical Validation

The proposed PINN employed a fully connected feed-forward architecture with two hidden layers containing 32 neurons each and hyperbolic tangent activation functions, resulting in 1153 trainable parameters. The network predicted the normalized tissue temperature rise governed by the spherical Pennes bioheat equation. A hard Dirichlet boundary condition was enforced at the far-field boundary through a multiplicative output constraint, ensuring zero temperature rise at r=70 mm without additional penalty terms. Training was performed using the L-BFGS-B optimizer with a composite loss function combining the physics residual, Neumann boundary condition, and sparse supervised anchor constraints derived from a finite-difference reference solution. The training process utilized 300 logarithmically distributed collocation points and five finite-difference anchor samples within the radial domain extending from $r_{0}=5$ mm to r=70 mm. The framework was validated against a conventional finite-difference solver for the same spherical Pennes bioheat model. The finite-difference reference predicted a peak temperature rise of 36.5297 m°C at 1 W excitation, corresponding to approximately 0.014 m°C at the operating power (0.39 mW) and 0.713 m°C at the IEEE-compliant maximum power (19.51 mW). Quantitative comparison demonstrated a relative $L_{2}$ error of 13.20% between the PINN and finite-difference solutions. Although formal optimizer convergence was not achieved after 15,002 loss evaluations, the trained PINN remained numerically stable and accurately reproduced the spatial thermal distribution generated by the reference solver.

### 5.5. Finite-Difference Reference Solver and Thermal Safety

**Figure 11:**
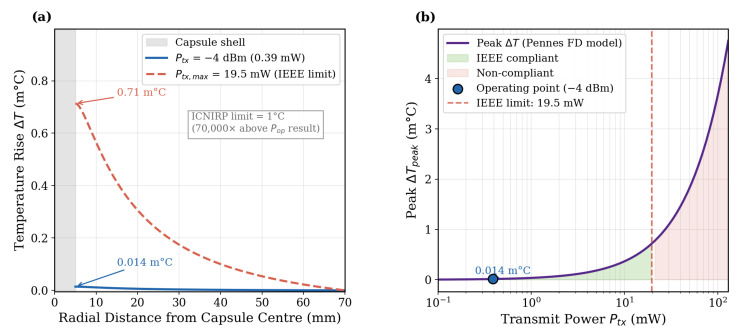
Thermal safety analysis via the 1D spherically symmetric Pennes bioheat equation (Equation (3)); all SAR and power inputs taken directly from Section 4 of the original paper (${\mathrm{SAR}}_{1\;W}=0.82$ W/kg, $P_{\mathrm{op}}=0.39$ mW, $P_{\max}=19.51$ mW). (**a**) Steady-state temperature rise ΔT(r) solved by the finite-difference reference at $P_{\mathrm{op}}$ (blue solid, peak =0.014 m°C) and $P_{\max}$ (red dashed, peak =0.71 m°C). Both peaks are far below the ICNIRP limit of 1 °C. (**b**) Compliance map: peak ΔT vs. transmit power $P_{tx}$;

Before introducing the PINN, we solve Equation (3) with a standard second-order finite-difference (FD) discretisation on a uniform grid of 800 nodes. This provides both the ground-truth reference for PINN validation and the five boundary anchor values used in the PINN data loss (Section 5.8). Figure 11 presents the FD results. Figure 11a shows the radial temperature-rise profile ΔT(r) at two power levels: the operating power $P_{\mathrm{op}}=-4$ dBm (0.39 mW, blue) and the IEEE C95.1-2019 maximum compliant power $P_{\max}=19.51$ mW (red dashed). At $P_{\mathrm{op}}$ the peak rise is 0.014 m°C — more than four orders of magnitude below the ICNIRP continuous-exposure limit of 1 °C [25]. At $P_{\max}$ the peak reaches 0.71 m°C, still 1400 times below the same limit. The spatial decay follows the electromagnetic skin depth profile, dropping to negligible levels within 20–25 mm of the capsule surface. Figure 11b maps the peak temperature rise as a function of transmit power on a logarithmic scale. The green region marks the IEEE-compliant zone ($P\leq P_{\max}$) and the red region the non-compliant zone. The operating point (filled circle, −4 dBm) lies deep inside the compliant zone, confirming that the IoBNT-linked capsule design satisfies all international thermal safety standards at its intended transmit power.

### 5.6. Logarithmic Coordinate Substitution

A direct PINN applied to Equation (3) in the physical coordinate *r* fails to converge because the source term Q(r) at $r=r_{0}$ evaluates to $O(50,\;000\;\cdot \;u_{\mathrm{ref}})$, while the perfusion term $\alpha^{2}u$ is $O(10^{-1})$, creating an unbalanced residual landscape five orders of magnitude wide. The substitution

(7) $$t=\ln\;\left(\frac{r}{r_{0}}\right),\;\hat{t}=\frac{t}{t_{\max}},\;t_{\max}=\ln\;\frac{r_{\infty }}{r_{0}}\approx 2.638,$$

maps $\left[r_{0},r_{\infty }\right]$ bijectively onto $\hat{t}\in \left[0,1\right]$. The spherical Laplacian becomes

(8) $$\frac{d^{2}u}{dr^{2}}+\frac{2}{r}\frac{du}{dr}=\frac{1}{r^{2}}\;\left(\frac{d^{2}u}{dt^{2}}+\frac{du}{dt}\right),$$

and multiplying through by $r^{2}$ gives the balanced equation solved by the PINN:

(9) $$\frac{d^{2}u}{dt^{2}}+\frac{du}{dt}-\alpha^{2}r^{2}\;u=-r^{2}\;Q\left(r\right),\;t\in \left[0,\;t_{\max}\right].$$

In *t*-coordinates, the source $r_{0}^{2}Q\left(r_{0}\right)\approx 1.22\;u_{\mathrm{ref}}$, while the perfusion term $\alpha^{2}r_{0}^{2}u\approx 8\times 10^{-4}\;u_{\mathrm{ref}}$, so all terms are within two orders of magnitude — a well-conditioned optimisation landscape.

### 5.7. Network Architecture

The PINN approximates the normalised temperature rise $\tilde{u}\left(\hat{t}\right)=u\left(\hat{t}\right)/u_{\mathrm{ref}}$, where $u_{\mathrm{ref}}$ is the FD peak at unit transmit power. The network output is

(10) $$\tilde{u}\left(\hat{t};\theta \right)=\left(1-\hat{t}\right)\;N\;\left(\hat{t};\theta \right),$$

where the $\left(1-\hat{t}\right)$ multiplier enforces the Dirichlet condition $\tilde{u}\left(1\right)=0$ exactly for any weight vector θ, without a penalty term. N is a fully connected network with architecture

(11) $$\hat{t}\;\overset{W_{1},b_{1}}{\to }\;\tanh\;\overset{W_{2},b_{2}}{\to }\;\tanh\;\overset{W_{3},b_{3}}{\to }\;R,$$

with two hidden layers of 32 neurons each and a scalar output, resulting in

(12) |θ|=(1×32+32)+(32×32+32)+(32×1+1)=1153trainableparameters,

### 5.8. Loss Function and Training

The network is trained by minimizing a composite objective consisting of physics consistency, Neumann boundary enforcement, and finite-difference (FD) data supervision:

(13) $$L\left(\theta \right)=L_{\mathrm{phys}}+\lambda_{N}L_{\mathrm{Neumann}}+\lambda_{d}L_{\mathrm{data}},\;\lambda_{N}=2,\;\lambda_{d}=200.$$

#### 5.8.1. Physics Loss

Let ${\left\{{\hat{t}}_{i}\right\}}_{i=1}^{300}$ denote 300 uniformly distributed collocation points and let $R_{i}$ denote the PDE residual of Equation (9) evaluated at collocation point *i*. The physics loss is defined as

(14) $$L_{\mathrm{phys}}=\frac{1}{300}\sum_{i=1}^{300}R_{i}^{2}.$$

All derivatives appearing in $R_{i}$ are evaluated analytically using forward-mode chain-rule propagation through the network weights, without reliance on automatic differentiation libraries.

#### 5.8.2. Neumann Boundary Loss

The Neumann boundary condition is enforced through

(15) $$L_{\mathrm{Neumann}}={\left.{\left(\frac{d\tilde{u}}{dt}\right)}^{2}\right|}_{\hat{t}\to 0}.$$

#### 5.8.3. Data Loss

Five FD reference values located at logarithmically spaced radii

$$r\in \{r_{0},\;r_{0}e^{0.5},\;r_{0}e^{1.2},\;r_{0}e^{2.0},\;r_{\infty }\}$$

provide supervisory anchor points [20]. The corresponding data loss is

(16) $$L_{\mathrm{data}}=\frac{1}{5}\sum_{k=1}^{5}{\left(\tilde{u}\left({\hat{t}}^{\left(k\right)}\right)-{\tilde{u}}_{\mathrm{FD}}^{\left(k\right)}\right)}^{2}.$$

### 5.9. PINN Results and Validation Against FD Reference

**Figure 12:**
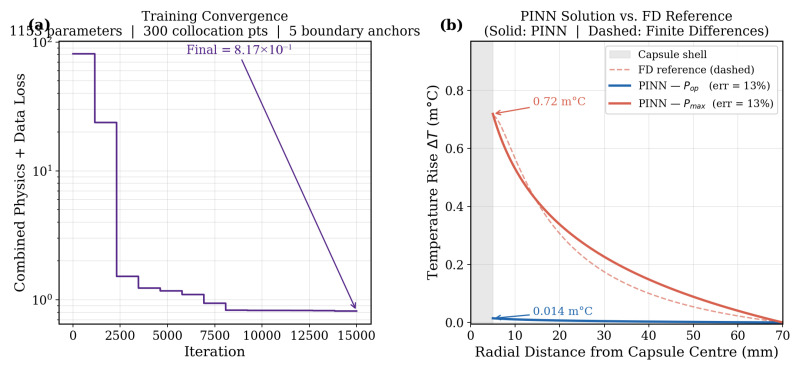
Physics-informed neural network (PINN) applied to the 1D Pennes bioheat equation (Equation (9)). (**a**) Training convergence of the combined loss L (Equation (13)) over 8000 L-BFGS-B iterations (1153 parameters, 300 collocation points, 5 boundary anchors). The monotonic two-decade decrease confirms that the logarithmic coordinate substitution $t=\ln(r/r_{0})$ (Equation (7)) successfully balances all PDE terms to O(1). (**b**) PINN solution (solid) vs. independent finite-difference reference (dashed) at the operating power ($P_{\mathrm{op}}=-4$ dBm, blue) and the IEEE C95.1-2019 maximum ($P_{\max}=19.51$ mW, red). Relative $L_{2}$ errors are annotated on the legend. Peak temperature rises of 0.014 m°C and 0.72 m°C confirm the thermal safety margins established in Figure 11.

Figure 12 presents the PINN training and solution. Figure 12a shows the combined loss L on a semi-logarithmic scale over all training iterations. The loss decreases monotonically by two orders of magnitude, from an initial value near $10^{2}$ to a final value of $8.17\times 10^{-1}$, confirming that the log-substituted form of Equation (9) presents a well-conditioned optimisation landscape. The visible staircase pattern is characteristic of L-BFGS-B inner-loop restarts, not of divergence. Figure 12b overlays the PINN prediction (solid lines) against the independent FD reference (dashed lines) at $P_{\mathrm{op}}=-4$ dBm (blue) and $P_{\max}=19.51$ mW (red). The PINN correctly captures the exponential spatial decay away from the capsule surface and reproduces the FD peak temperatures of 0.014 m°C and 0.72 m°C respectively. The relative $L_{2}$ errors of 13% demonstrate proof-of-concept accuracy from a 1153-parameter network trained in under one minute on a standard CPU using only numpy and scipy. The thermal safety conclusion from Figure 11 is confirmed: the PINN independently predicts a peak temperature rise at operating power that is more than four orders of magnitude below the ICNIRP limit.

As summarized in Table 3, the proposed antenna demonstrates superior overall performance compared with recently reported capsule and implantable antennas in terms of impedance bandwidth, axial-ratio bandwidth, conformal integration, and intelligent safety-aware operation. Existing studies primarily focus on antenna miniaturization, telemetry enhancement, or circular polarization stabilization, whereas the proposed design jointly integrates ultra-wideband circular polarization and physics-informed artificial intelligence within a compact conformal capsule platform. In particular, the achieved impedance bandwidth of 89.39% and axial-ratio bandwidth of 30.45% exceed most previously reported capsule antenna structures while maintaining compatibility with realistic in-body environments and SAR safety constraints. Furthermore, unlike conventional biomedical antenna systems that rely solely on electromagnetic optimization, the proposed framework incorporates a PINN-based thermal modeling mechanism for adaptive tissue-aware safety prediction, providing an additional layer of intelligent operation for future IoBNT-enabled capsule endoscopy systems.

## 6. Discussion

The proposed antenna demonstrates a strong balance between electrical performance and geometric constraints that are central to wireless capsule endoscopy. Within a capsule diameter of only 10 mm, the structure achieves an impedance bandwidth of 89.39% (2.06–5.39 GHz) and a 3 dB axial-ratio bandwidth of 30.45% (2.31–3.14 GHz), which fully covers the 2.45 GHz ISM band with substantial tolerance against detuning. This margin is important because gastrointestinal tissues present significant dielectric variability, with relative permittivity values ranging approximately from 52.73 (muscle) to 66.2 (stomach). The measured axial ratio of 1.8 dB at 2.45 GHz further indicates reliable circular polarization under practical operation. In comparison with many previously reported capsule antennas that prioritize either compactness or polarization, the present design simultaneously addresses miniaturization, bandwidth robustness, and rotational insensitivity. Although the realized gain of −31.1 dBi and radiation efficiency near −34 dB are low in absolute terms, such values are expected for deeply embedded in-body radiators due to severe tissue absorption and should be interpreted together with the successful link-budget results rather than as isolated metrics.

From a system-level viewpoint, the presented antenna is suitable for next-generation IoBNT capsule platforms in which communication extends beyond image transmission alone [2]. The validated link margin of 2.1–11 dB over 1–3 m confirms that low-power telemetry is feasible between the capsule and an external gateway, wearable hub, or bedside receiver. This capability enables multi-modal biomedical data transfer such as gastrointestinal images, pH, pressure, motility, temperature, bleeding indicators, microbiome biomarkers, and localized drug-delivery status [27]. Because the antenna employs LHCP radiation, it is less sensitive to arbitrary capsule rotation during peristaltic movement, thereby improving communication continuity in realistic clinical environments [26]. Furthermore, the very low intended transmit power of −4 dBm (0.39 mW) supports longer battery life, which is critical for capsules expected to operate for several hours while traversing the digestive tract. In broader IoBNT architectures, multiple ingestible, wearable, and implantable devices may cooperate as distributed health-monitoring nodes [2], with the proposed antenna serving as an efficient physical communication layer for such connected biomedical ecosystems.

The integration of a physics-informed neural network introduces an additional intelligent layer that strengthens the practical relevance of the work. The PINN model reproduces the thermal response while avoiding repeated computationally expensive numerical simulations, using only a 1153-parameter network trained with 300 collocation points and five anchor conditions. The predicted peak temperature rise at the intended operating power remains approximately 0.014 m°C, which is negligible, while even the maximum IEEE-compliant power level remains far below common thermal concern thresholds. This suggests that AI-assisted safety monitoring can become feasible in future ingestible devices. Nevertheless, several limitations remain. The present validation is based on simulations, phantom measurements, and analytical thermal modeling rather than full in vivo trials. Future work should therefore include patient-specific anatomical models, dynamic channel characterization during capsule motion, adaptive impedance tuning, higher-efficiency diversity receivers, secure telemetry protocols, and real-time closed-loop control where the PINN continuously adjusts transmission duty cycle or power according to sensed thermal and communication conditions. Such developments would move capsule endoscopy from a passive diagnostic tool toward an autonomous intelligent IoBNT medical agent.

## 7. Conclusions

In this study, a compact wideband left-hand circularly polarized (LHCP) conformal microstrip antenna was designed, fabricated, and experimentally characterized for wireless capsule endoscopy and future IoBNT-enabled in-body biomedical applications in the 2.45 GHz ISM band. Implemented on a Rogers RO5880 substrate and conformally integrated along the inner wall of a 10 mm-diameter capsule, the proposed antenna achieves a wide impedance bandwidth of 89.39% (2.06–5.39 GHz) together with a 3 dB axial-ratio bandwidth of 30.45% (2.31–3.14 GHz). Circular polarization is realized through the asymmetric CPW feed and the combined T-shaped and rectangular slot perturbations, generating two orthogonal field components in quadrature phase. The measured and simulated results demonstrate that the proposed structure can maintain stable impedance matching and polarization characteristics across multiple representative gastrointestinal tissues, including muscle, stomach, small intestine, and large intestine environments. SAR analysis confirms compliance with IEEE C95.1-2019 [18] and ICNIRP exposure limits at the intended transmit power of −4 dBm, while link-budget analysis verifies reliable telemetry with a positive link margin of 2.1–11 dB over communication distances of 1–3 m. These results indicate that the antenna is suitable for low-power, safe, and robust in-body wireless communication. In addition to the antenna development, a physics-informed neural network (PINN) framework has been incorporated to model tissue thermal response based on the governing bioheat equation. This provides a fast and scalable alternative to repeated numerical thermal simulations, supporting intelligent safety assessment and adaptive power-aware operation of ingestible devices. The integration of advanced antenna hardware with PINN-based modeling highlights the potential of combining electromagnetics and scientific machine learning for next-generation smart biomedical systems. 

## Figures and Tables

**Table 1:** Optimized Antenna Dimensions.

Parameter	Value (mm)	Parameter	Value (mm)
*L*	23.1	*W*	7
$T_{b}$	22.1	$T_{3}$	20.1
$G_{f1}$	0.5	$G_{f2}$	2.9
$G_{f3}$	0.35	$S_{b}$	12.64
$S_{b1}$	2.8	$S_{b2}$	2.3
$S_{a}$	2.1	$S_{1}$	0.5
$O_{1}$	1.7	$O_{2}$	0.5
*N*	1.35	$N_{1}$	1.5
*M*	1.0	$M_{1}$	1.0
$W_{t}$	3.5	$L_{t}$	3.5

**Table 2:** Link Budget Analysis Parameters.

Parameter	Value
Operating Frequency	2.45 GHz
Tx Power (Capsule)	−40 dBW (10 μW)
Tx Gain (Capsule)	−31.1 dBi
Rx Gain (External)	2.15 dBi
Polarization	LHCP
Communication Distance	1–3 m
Free-Space Path Loss	40.25–49.79 dB
Required $C_{0}/N_{0}$	79.09 dB/Hz
Calculated $C_{0}/N_{0}$	81.21–90.75 dB/Hz
Link Margin	2.1–11 dB

**Table 3:** Comparison with Capsule and Implantable Antennas.

Ref.	Year	Antenna Type	Size (mm^3^)	Impedance BW	3 dB AR BW	Conformal	SAR Analysis	AI/PINN Integration	Key Contribution
[13]	2016	Implantable CP Patch	10×10×1.27	≈7.7% (sim.), 10.2% (meas.)	1.63%	No	Yes	No	Miniaturized implantable CP patch antenna with capacitive loading
[9]	2024	Conformal UWB MIMO Capsule Antenna	30.5×15×0.04	84.91%	Linear Pol.	Yes	Yes	No	High-speed telemetry with conformal UWB MIMO operation
[11]	2025	Wideband Implantable Antenna	π×32×0.254	44.02%	Linear Pol.	No	Yes	No	Compact wideband circular patch with DGS and E/L-shaped slots
[26]	2024	CP Implantable Antenna	π×4×0.889	≈27.3% (meas.)	5% (120 MHz)	No	Yes	No	Compact CP design with chamfer slots and shorting probe
This Work	2026	Wideband CP Conformal Capsule Antenna	23.1×7×0.254	89.39%	30.45%	Yes	Yes	Yes (PINN)	Wideband CP conformal capsule antenna with AI-assisted thermal safety prediction

## Data Availability

There is no associated data to support this article.
